# Reverse engineering gene regulatory network based on complex-valued ordinary differential equation model

**DOI:** 10.1186/s12859-021-04367-2

**Published:** 2021-09-20

**Authors:** Bin Yang, Wenzheng Bao, Wei Zhang, Haifeng Wang, Chuandong Song, Yuehui Chen, Xiuying Jiang

**Affiliations:** 1 School of Information Science and Engineering, Zaozhuang University, Zaozhuang, 277160 China; 2 School of Information and Electrical Engineering, Xuzhou University of Technology, Xuzhou, 221018 China; 3 School of Information Science and Engineering, University of Jinan, Jinan, 250022 China

**Keywords:** Gene regulatory network, Complex-valued ordinary differential equation, Grammar-guided genetic programming, Firefly algorithm

## Abstract

**Background:**

The growing researches of molecular biology reveal that complex life phenomena have the ability to demonstrating various types of interactions in the level of genomics. To establish the interactions between genes or proteins and understand the intrinsic mechanisms of biological systems have become an urgent need and study hotspot.

**Results:**

In order to forecast gene expression data and identify more accurate gene regulatory network, complex-valued version of ordinary differential equation (CVODE) is proposed in this paper. In order to optimize CVODE model, a complex-valued hybrid evolutionary method based on Grammar-guided genetic programming and complex-valued firefly algorithm is presented.

**Conclusions:**

When tested on three real gene expression datasets from *E.* *coli* and Human Cell, the experiment results suggest that CVODE model could improve 20–50% prediction accuracy of gene expression data, which could also infer more true-positive regulatory relationships and less false-positive regulations than ordinary differential equation.

## Background

The aim of gene regulatory network (GRN) research is to explain comprehensively the generation process and complex regulatory relationships of genes and their products in biological tissues from the perspective of system scale [1–3]. GRN is an important way to cope with various internal and external stimuli by synergizing the functions of genes. GRN is a complex continuous and dynamic system, in which gene regulation is a dynamic event that changes with time and environment [4–6]. Gene expression data could reflect the regulatory orders of genes and the important information of regulatory objects [7]. With the development of DNA microarray or chip technology, a large amount of gene expression profiles have been generated, which provide a condition for identifying gene regulatory networks using computational models [8–10].

Some computational approaches have been developed to describe biology networks [11–17], especially the regulatory relationships between genes with gene expression time series, such as directed graph [18], Boolean network [3, 19], Bayesian network [20, 21], differential equation [22], neural network [23–25], stochastic equation [26, 27], etc. Differential equation model is more conducive to describing the concentration evolution of biological macromolecules such as RNA and protein over time. Therefore, this model has been widely utilized in pharmacokinetics, enzymology and gene regulatory network construction [28–33]. For each target gene, its corresponding differential equation is identified, in which the dependent variables (genes) indicate the corresponding regulatory factors of target gene. Hohm and Zitzler resolved the issue of how to optimize the parameters of ordinary differential equation (ODE) for GRN inference [34]. Tian et al. proposed stochastic delay differential equations to describe the time-delayed regulatory relationships in GRN [35]. Gebert and Jong proposed piecewise linear differential equation to identify the most relevant regulating interactions in GRN [36, 37]. Zhang et al. proposed single-index ODE model and clipped absolute deviation penalty penalized function to infer network structure [38]. Matsumoto et al. proposed ODE models to identify GRN with single-cell RNA-Seq data [39]. Some special nonlinear ODE models also have been proposed to infer GRN, such as S-system model [40–42].

Recently the complex versions of many models have been proposed due to their potential to optimize more easily, better generalization characteristics, faster learning and higher noise-robust memory mechanisms [43–45]. You and Hong presented multilayer feedforward neural network (MFNN) with complex-valued activation function to model QAM signals of different constellation sizes [46]. Deng et al. also proposed complex-valued radial basis function neural network (CVRBFNN) to deal with QAM signals and the results showed that CVRBFNN performed better than functional link artificial neural network [47]. Hu et al. discussed the global stability of the complex-valued and delayed version of recurrent neural network [48]. Trabelsi et al. proposed complex-valued convolutional feed-forward networks [49]. Yang et al. proposed a novel complex-valued method based on mathematical expression to resolve real-valued prediction and classification problems [50].

In order to forecast gene expression data and identify GRN accurately, complex-valued ordinary differential equation (CVODE) is presented in this paper. Grammar-guided genetic programming (GGGP) is utilized to evolve the structure of CVODE and complex-valued firefly algorithm (CFA) is proposed to search the optimal complex-valued parameters of model. Three real gene expression datasets are applied to test the inference performances of our proposed methods.

## Results and discussion

Three real gene expression data sets are utilized to test the performance of our proposed method. All the real gene expression data need to be normalized, which is defined as follows.

1 $$g^{\prime} = \frac{{g - g_{\min } }}{{g{}_{\max } - g_{\min } }}$$

where $g_{\min }$ and $g_{\max }$ are the minimum and maximum of dataset, respectively.

In our experiment, ODE and CVODE models are both applied to predict gene expression data and infer gene regulatory networks with three real gene expression datasets. Our proposed hybrid evolutionary algorithm is utilized to infer ODE and CVODE models and the parameters are the same. In GGGP, population size is set to 50, crossover rate is set to 0.9 and mutation rate is set to 0.1. In CFA, population size is set as 100, and step size is set as 0.02. The parameters are selected according to previous research results [51–53]. Sensitive ($S_{n}$) and Specificity ($S_{p}$) are utilized to evaluate the inferred gene regulatory networks. $S_{n}$ represents the proportion of the positive samples to be identified correctly in all positive ones. $S_{p}$ denotes the proportion of the negative samples to be identified correctly in all negative ones.

### SOS DNA repair network

**Fig. 1:**
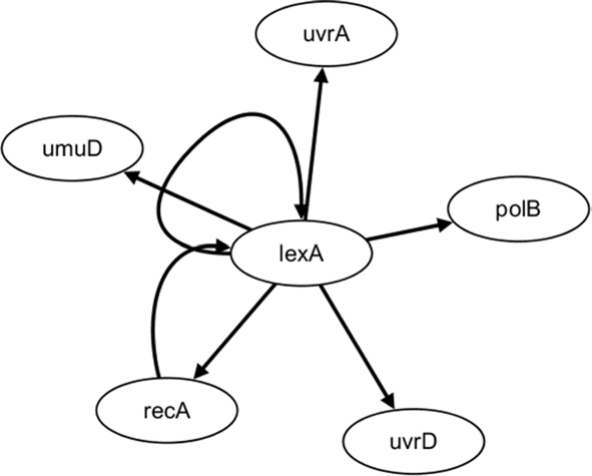
SOS DNA repair network

SOS DNA damage repair network is selected as the first real gene regulatory network, which contains six main genes (**uvrD**, **lexA**, **umuD**, **recA**, **uvrA** and **polB**). The real structure of SOS DNA damage repair network is depicted in Fig. 1. The gene expression data used in this experiment are from Uri Alon [54, 55]. The DNA repair process of *E. coli* is observed by ultraviolet irradiation. The expression levels of six genes at different time points are measured.

The datasets from the first three experiments are utilized to optimize the CVODE models. The last dataset is utilized to test. With hybrid evolutionary algorithm the corresponding CVODE models of six genes are described as follows. Variables $x_{1} ,x_{2} , \ldots ,x_{6}$ represent six genes: **uvrD**, **lexA**, **umuD**, **recA**, **uvrA** and **polB**, respectively.

2 $$\begin{aligned} \frac{{dx_{1} }}{dt} & = {( - 0}{\text{.1523 - 0}}{.1507 }i{)}\frac{{x_{1} }}{{x_{5} }}{ + ( - 2}{\text{.4023 + 0}}{.0498 }i{)} \times {(}x_{{1}} { - }x_{{6}} {) + (0}{\text{.2349 + 0}}{.1709 }i{)}x_{{2}} \\ \frac{{dx_{2} }}{dt} & = {(0}{\text{.3382 + 3}}{.3836 }i{)} \times {(1 - }x_{{1}} {) + (4}{\text{.4767 + 0}}{.0309 }i{)}x_{{6}} { + (0}{\text{.0773 - 0}}{.0837 }i{)} \times {\text{cos(}}x_{{4}} { - }x_{{2}} {)} \\ \frac{{dx_{3} }}{dt} & = {(0}{\text{.222 + 0}}{.8159 }i{)}x_{{3}} x_{{1}} { + (0}{\text{.906 + 1}}{.638 }i{\text{)cos(}}x_{{3}} {) + (0}{\text{.6876 - 0}}{.514 }i{)} \times {(}x_{{2}} { + }x_{{1}} {)} \\ \frac{{dx_{4} }}{dt} & = {(4}{\text{.396 + 2}}{.799 }i{)} \times {(2}x_{{2}} { - }x_{{6}} {) + ( - 9}{\text{.0003 + 2}}{.4687 }i{)} \times {(}\frac{{x_{1} }}{{x_{4} }}{)}^{2} { + ( - 7}{\text{.023 + 10}}{.339 }i{)} \\ & \quad \times {\text{(cos(}}x_{{3}} {) - }x_{{3}} {) + ( - 3}{\text{.2559 + 2}}{.3771 }i{)} \times {(}x_{{4}} { - }\frac{{x_{6} }}{{x_{4} }}{) + (0}{\text{.4655 - 0}}{\text{.1199 i)cos(}}x_{{1}} { - }x_{{5}} {)} \\ \frac{{dx_{5} }}{dt} & = {( - 0}{\text{.2329 + 0}}{.7256 }i{\text{)cos(sin(}}x_{{1}} {)) + (0}{\text{.7061 + 0}}{.0325 }i{\text{)cos(}}x_{{5}} { - }x_{{2}} {) + (0}{\text{.0241 - 0}}{.5961 }i{\text{)cos(cos(}}x_{{3}} {))} \\ \frac{{dx_{6} }}{dt} & = {(0}{\text{.0461 + 0}}{.0237 }i{)}x_{{3}} x_{{2}} { - (0}{\text{.2843 + 0}}{.0345 }i{)}x_{6}^{3} \\ \end{aligned}$$

**Fig. 2:**
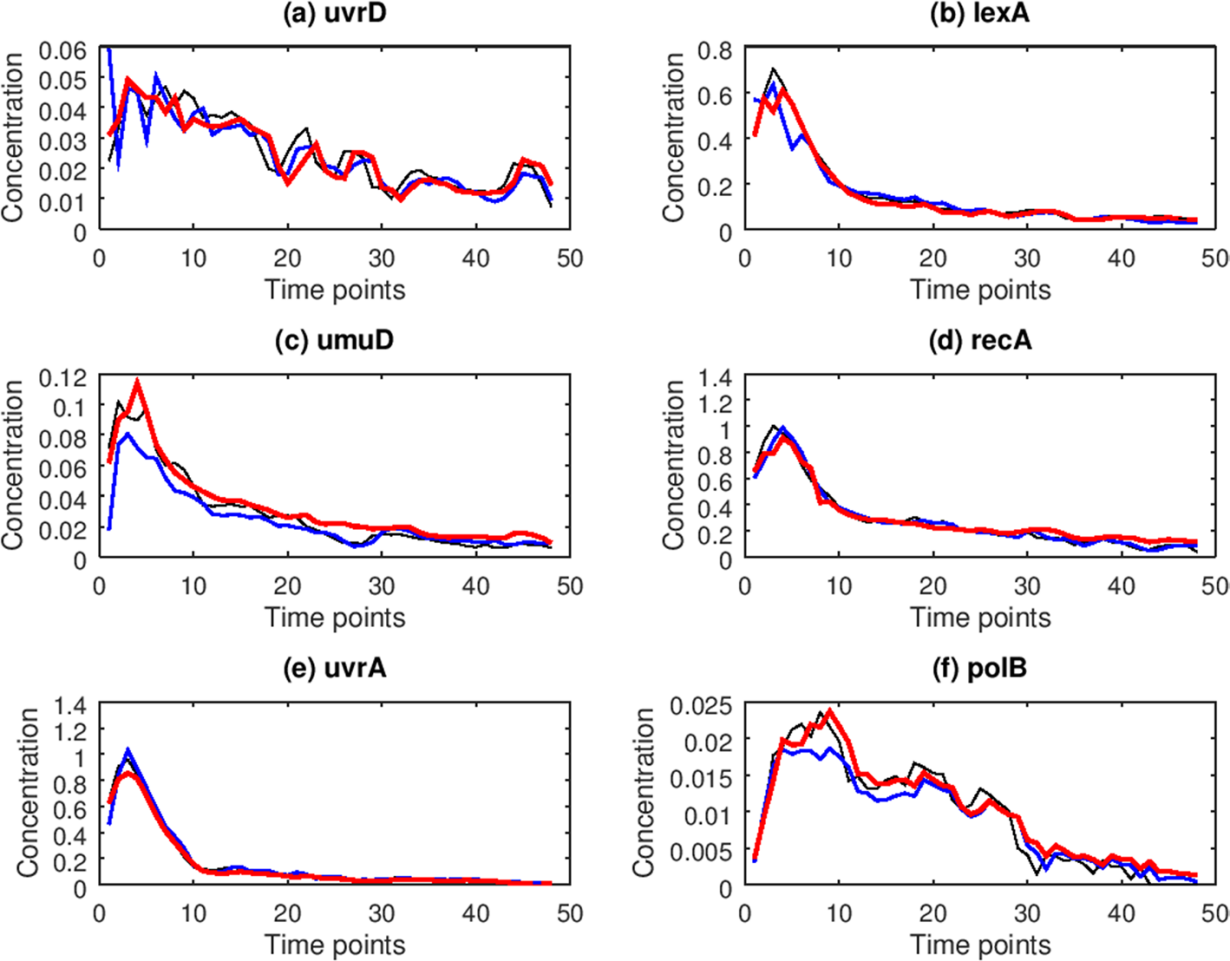
The prediction results of 6 genes in SOS network. The black lines represent the real gene expression data, while the blue lines and red lines denote the forecasting results by ODE and CVODE models, respectively

ODE models are also utilized to infer SOS network. The predicted results of ODE and CVODE for six genes are depicted in Fig. 2. From Fig. 2, it could be seen that the results of CVODE predicted are closer to real ones than ODE. In order to compare clearly, the prediction RMSE values of ODE and CVODE for six genes are listed in Table 1, which reveals that CVODE model has smaller prediction RMSE values than ODE for six genes. On average CVODE could decrease 34.4% RMSE value.

**Table 1:** Prediction RMSE values of ODE and CVODE for six genes

Genes	ODE	CVODE
**uvrD**	0.014345	**0.007601**
**lexA**	0.075643	**0.072955**
**umuD**	0.017476	**0.008536**
**recA**	0.164121	**0.076576**
**uvrA**	0.168299	**0.122481**
**polB**	0.004601	**0.003229**
Averaged	0.074081	**0.048563**

The ‘bold values’ represent the better results between two methods

**Fig. 3:**
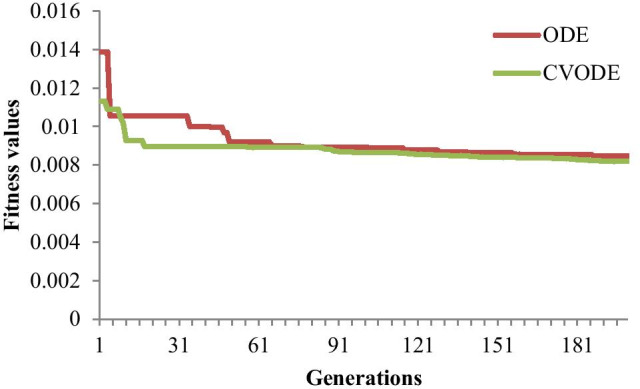
Fitness values vs generations

In order to test the modeling performance of CVODE further, the fitness values of ODE and CVODE against generations are plotted in Fig. 3. From Fig. 3, we can see that the hybrid evolutionary algorithm could search the optimal CVODE model with about 20 generations, while the optimal ODE model is found with about 50 generations. Thus CVODE model has smaller fitness value and gains the optimal solution with fewer generations than ODE model.

**Fig. 4:**
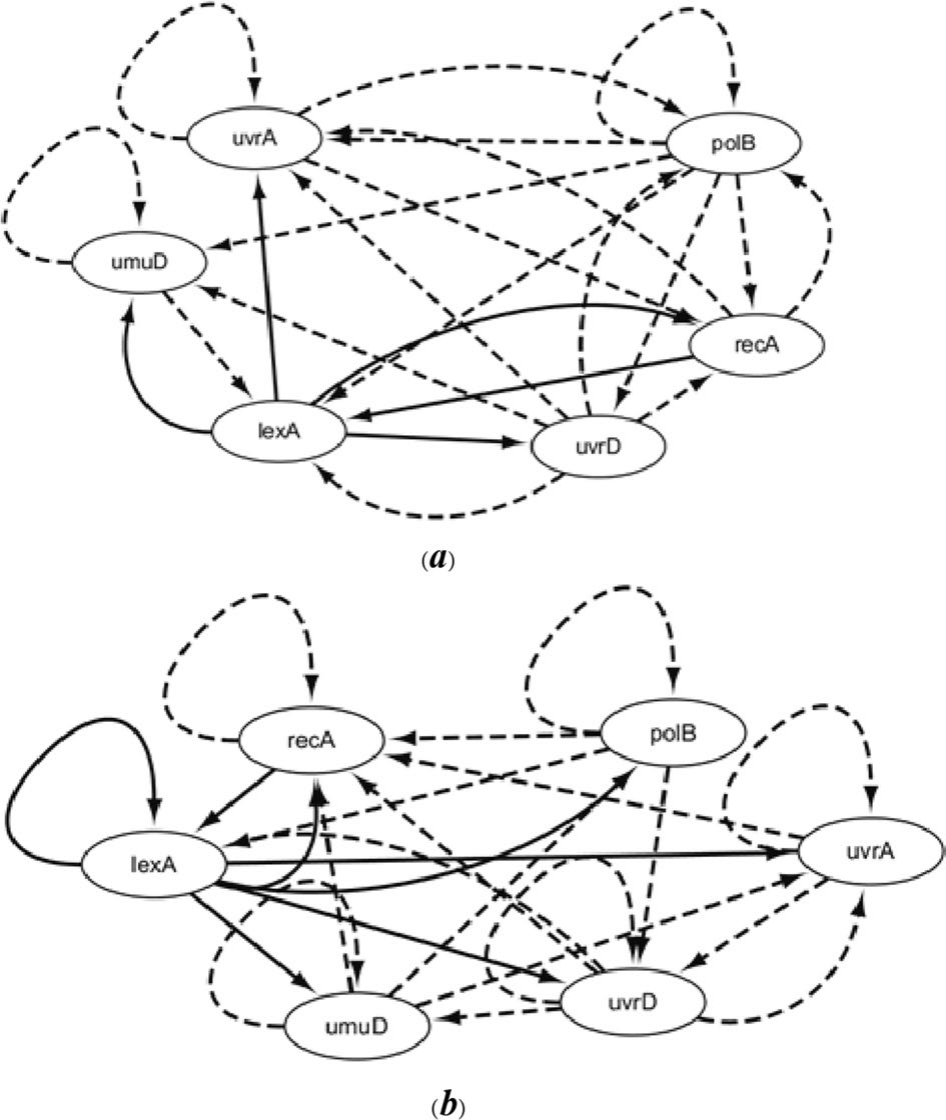
The construction of SOS network by ODE (**a**) and CVODE (**b**). The solid lines denote the real regulations, while the dotted lines represent the false relationships

The inferred SOS networks by ODE and CVODE are depicted in Fig. 4. Compared Fig. 1 with Fig. 4, it could be seen that CVODE model could identify 7 true-positive (real) regulations and 17 false-positive regulatory relationships, while ODE model could identify 5 true-positive relationships and 18 false-positive relationships. The performances of $S_{n}$ and $S_{p}$ of SOS networks inferred by ODE and CVODE are listed in Table 2. In terms of $S_{n}$, CVODE is 39.99% higher than ODE. In terms of $S_{p}$, our method is 9.1% higher than ODE. The results reveal that our method could identify all true-positive regulatory relationships and less false-positive regulations than ODE model.

**Table 2:** Performances of ODE and CVODE for construction of SOS network

Methods	$S_{n}$	$S_{p}$
CVODE	**1**	**0.4138**
ODE	0.7143	0.3793

The ‘bold values’ represent the better results between two methods

### Human cell time-series data

Cell cycle refers to the whole the process from the beginning of one cell division to the end of the next division. The second real gene expression time series data are from the genes periodically expressed, which were supplied by Whitfield [56]. The experiment includes five periods and 1134 genes. The sub dataset extracted in this part includes five genes and 46 time points, in which the first 40 time points are utilized to search the optimal CVODE models and the rest time points are utilized to test the performance. By applying our method, we have obtained the following CVODE models. Variables $x_{1} ,x_{2} , \ldots ,x_{5}$ represent five genes, respectively.

3 $$\begin{aligned} \frac{{dx_{1} }}{dt} & = {(0}{\text{.2366 + 4}}{.2126 }i{)} \times {(}x_{{5}} { + }x_{{2}} {) - (1}{\text{.0667 - 0}}{.0343 }i{)}x_{4}^{2} { - (4}{\text{.9572 - 2}}{.8702 }i{)} \\ & \;\;\;x_{{3}} x_{{5}} { + (6}{\text{.5323 - 0}}{.7549 }i{)}x_{{3}} { - (4}{\text{.3955 + 0}}{.5311 }i{)} \times {(}x_{{1}} { - }x_{{4}} {)} \\ \frac{{dx_{2} }}{dt} & = {(1}{\text{.5138 + 11}}{.009 }i{)} \times {(}x_{{5}} { - }x_{{4}} {) - (6}{\text{.201 + 5}}{.4258 }i{)}x_{{3}} { + (2}{\text{.0535 + 0}}{.2076 }i{)} \\ & \;\;\; \times {(}x_{{1}} { + }x_{{3}} {) - (3}{\text{.7539 - 4}}{.6864 }i{\text{)cos(}}x_{{3}} {)} \\ \frac{{dx_{3} }}{dt} & = {(2}{\text{.7072 + 3}}{.5088 }i{)}\frac{{x_{4} }}{{x_{1} }}{ - (3}{\text{.3836 + 0}}{\text{.1783 i)}} \times {(}x_{{4}} { - }x_{{3}} {) - (0}{\text{.941 + 0}}{.5496 }i{)} \\ & \;\;\; \times {\text{cos(}}x_{{3}} {) + (2}{\text{.8053 + 3}}{.0456 }i{)}x_{{4}} \\ \frac{{dx_{4} }}{dt} & = {( - 0}{\text{.029 - 2}}{.7968 }i{\text{)sin(}}x_{{5}} {) + (4}{\text{.5367 + 5}}{.986 }i{\text{)sin(}}x_{{1}} {) - (4}{\text{.9878 + 0}}{.757 }i{)} \times {(}x_{{4}} { - }x_{{3}} {)} \\ \frac{{dx_{5} }}{dt} & = {( - 4}{\text{.0444 + 0}}{.7446 }i{)} \times {(}x_{{1}} { - }x_{{4}} {) - (0}{\text{.9768 + 2}}{.0349 }i{)} \times {(}x_{{3}} { - }x_{{2}} {) + (3}{\text{.5815}} \\ & \;\;\;{ + 1}{\text{.2342 }}i{\text{)sin(}}x_{{1}} {) + ( - 1}{\text{.0076 + 3}}{.716 }i{)} \times {(}x_{{5}} { - }x_{{1}} {) + ( - 1}{\text{.0549 - 1}}{.8469 }i{)} \times {2}x_{{2}} \\ \end{aligned}$$

**Fig. 5:**
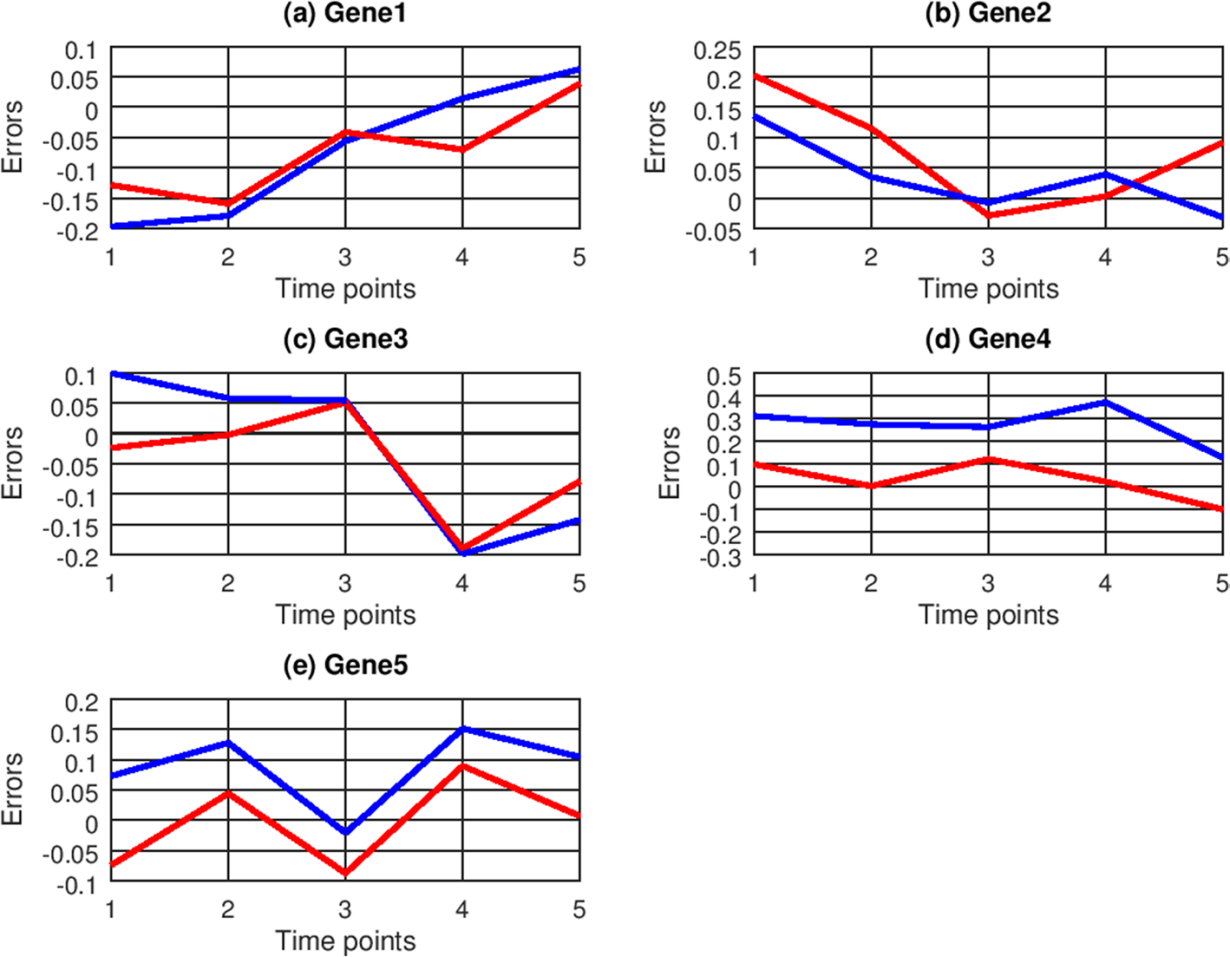
Prediction errors of ODE (blue lines) and CVODE (red lines) with human cell time-series data

The prediction errors of ODE and CVODE are depicted in Fig. 5, which reveals that the prediction errors of CVODE are closer to zero than ones of ODE. The predicted RMSE values of ODE and CVODE are listed in Table 3. For the predicted results of five genes, CVODE has smaller prediction RMSE values than ODE, which show that CVODE could more accurately predict gene expression data. On average CVODE could decrease 49.07% RMSE value.

**Table 3:** Prediction RMSE values of ODE and CVODE for six genes

Genes	ODE	CVODE
Gene 1	0.227851	**0.125126**
Gene 2	0.11358	**0.066308**
Gene 3	0.363917	**0.123348**
Gene 4	0.340177	**0.2797**
Gene 5	0.328782	**0.10551**
Averaged	0.274861	**0.139998**

The ‘bold values’ represent the better results between two methods

### *E. coli* database

**Fig. 6:**
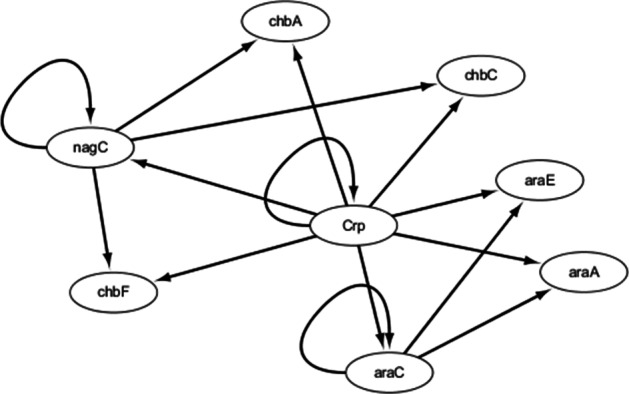
The real *E.* *coli* sub network

The third real gene expression dataset is extracted from polymicrobial probe database (version 4 build 6), which contains 4297 genes and 907 biology experiments [57]. The used dataset contains the first 35 experiments, in which the first 30 time points are utilized to search the optimal CVODE models and the rest time points are utilized as testing data. Eight genes (**Crp**, **araC**, **nagC**, **chbC**, **araE**, **araA**, **chbA** and **chbF**) are chosen and the real network structure is from RegulonDB [58] containing 15 regulations, which is described in Fig. 6.

Through our proposed hybrid evolutionary method, we could obtain the CVODE models as Eqs. (4). Variables $x_{1} ,x_{2} , \ldots ,x_{8}$ represent genes **Crp**, **araC**, **nagC**, **chbC**, **araE**, **araA**, **chbA** and **chbF**, respectively.

4 $$\begin{aligned} \frac{{dx_{1} }}{dt} & = {( - 4}{\text{.1015 + 5}}{.371 }i{)} \times {(}x_{{8}} { + }\frac{{x_{{1}} }}{{x_{{8}} }}{) + (2}{\text{.5219 - 0}}{.3343 }i{\text{)cos(}}x_{5}^{2} {) + (2}{\text{.0703 + 2}}{.9986 }i{)} \times {(}x_{{8}} { + }x_{{7}} \times x_{{8}} {)} \\ \frac{{dx_{2} }}{dt} & = {( - 7}{\text{.9767 + 9}}{.8189 }i{)}x_{7}^{2} { + (5}{\text{.5059 - 5}}{.2522 }i{)}x_{{2}} x_{{7}} { + (3}{\text{.5167 + 10}}{.3982 }i{)} \times {(}x_{2} { - }x_{{6}} {)} \\ & \;\;\;{ - (16}{\text{.939460 - 13}}{.448812 }i{)} \times {(}x_{{6}} { - }x_{{1}} {)} \\ \frac{{dx_{3} }}{dt} & = {( - 0}{\text{.1369 + 1}}{.0964 }i{\text{)cos(}}x_{{2}} {) + (1}{\text{.5904 + 0}}{.2761 }i{)}x_{3}^{2} { + (0}{\text{.2941 + 1}}{\text{.5517 i)}}x_{{2}} x_{{3}} { + } \\ & \;\;\;{ - (2}{\text{.4638 + 1}}{.6326 }i{)}\frac{{x_{{1}} }}{{x_{{8}} }}{ + (0}{\text{.8683 + 0}}{.6514 }i{)}\frac{{x_{{8}} }}{{x_{{2}} }} \\ \frac{{dx_{4} }}{dt} & = { ( - 5}{\text{.0047 - 0}}{.6521 }i{\text{)cos(sin(}}x_{{8}} {)) + (0}{\text{.79 + 0}}{.6943 }i{\text{)cos(}}x_{{3}} { - }x_{{1}} {) - (3}{\text{.9688 + 0}}{.779 }i{)} \\ & \;\;\; \times {(2}x_{{1}} { - }x_{{7}} {) + (1}{\text{.9222 + 0}}{.8736 }i{)}\frac{{\cos (x_{{4}} )}}{{x_{{4}} }}{ + (3}{\text{.234 - 0}}{.0632 }i{)}x_{{1}} \\ \frac{{dx_{5} }}{dt} & = {( - 1}{\text{.9009 - 0}}{.8821 }i{\text{)cos}}^{2} {(}x_{{5}} {) + (0}{\text{.5571 + 0}}{.9842 }i{)(}\frac{{x_{{2}} }}{{x_{{5}} }}{)}^{2} { + (2}{\text{.0521 - 0}}{.7683 }i{)}x_{{1}} \\ \frac{{dx_{6} }}{dt} & = {( - 0}{\text{.648 - 0}}{.2905} i{)}x_{{6}} { + ( - 2}{\text{.2871 + 0}}{.5342 }i{)}\frac{{x_{{4}} }}{{x_{{8}} }}{ - (1}{\text{.3399 + 4}}{.0571 }i{)}\frac{{x_{{6}} }}{{x_{{3}} }} \\ & \;\;\;{ - (0}{\text{.1901 + 2}}{.2337 }i{)}x_{{1}}^{2} { + (2}{\text{.1551 - 0}}{.3921 }i{)} \times {(}x_{{5}} { - }x_{{2}} {)} \\ \frac{{dx_{7} }}{dt} & = {( - 3}{\text{.1357 - 1}}{.2992 }i{\text{)cos(}}x_{{1}} {) + ( - 1}{\text{.8814 + 2}}{.4155 }i{)}x_{{3}}^{2} { + (0}{\text{.7295 + 0}}{.7151 }i{)}x_{{4}} x_{{1}} \\ & \;\;\;{ + ( - 1}{\text{.4544 - 0}}{.4791 }i{\text{)sin(}}x_{{4}} {)} \\ \frac{{dx_{8} }}{dt} & = {( - 1}{\text{.7613 - 3}}{.4795 }i{)} \times {(}x_{{1}} { - }x_{{6}} {) + (0}{\text{.1275 + 1}}{.415 }i{)}x_{{1}} { + (3}{\text{.3253 - 4}}{.9547 }i{\text{)sin(}}x_{8} {)} \\ & \;\;\;{ + ( - 2}{\text{.8165 + 1}}{.5682 }i{)}\frac{{x_{{7}} }}{{x_{{1}} }}{ + ( - 0}{\text{.6489 + 1}}{.2929} i{)}x_{6}^{2} \\ \end{aligned}$$

**Fig. 7:**
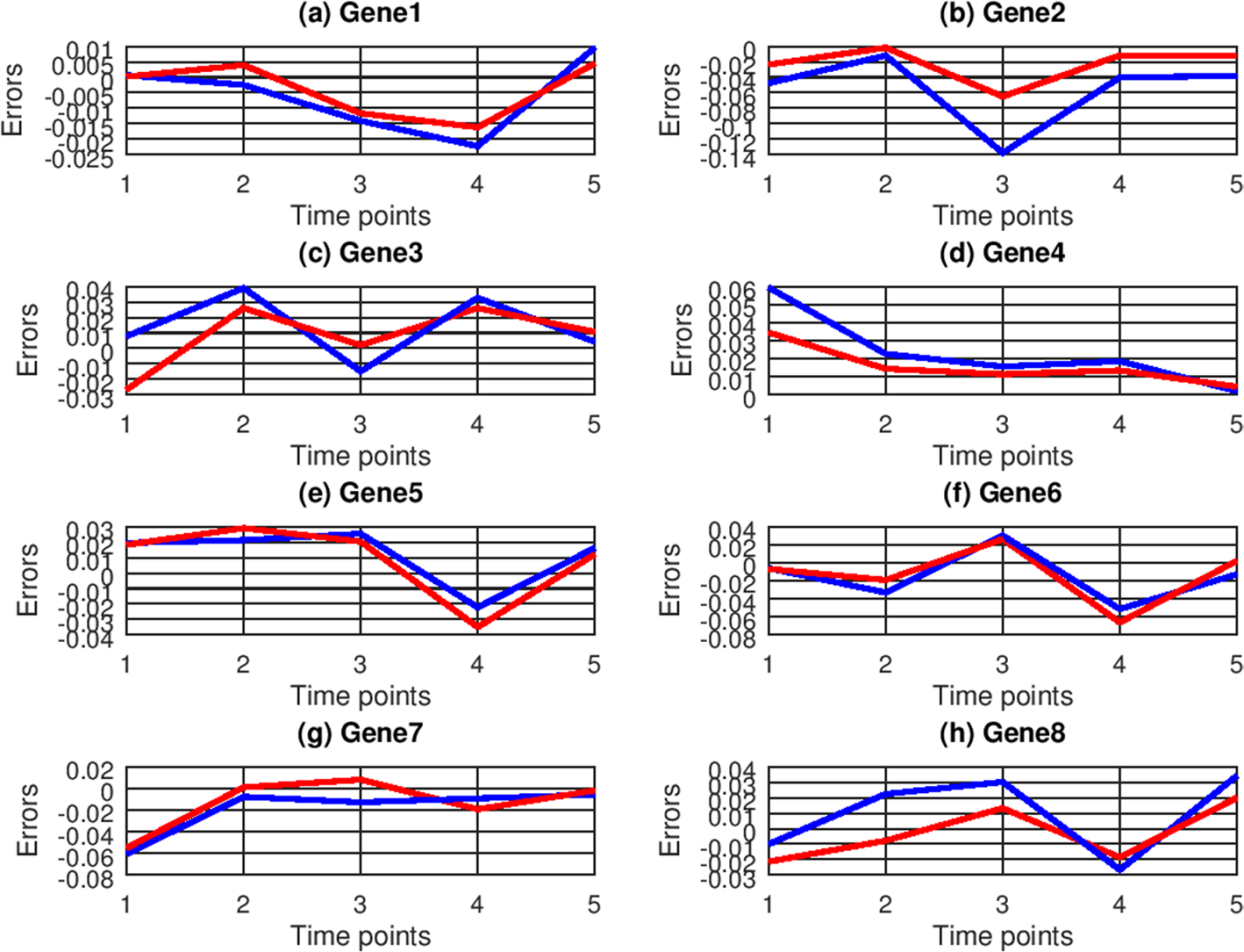
Prediction errors of ODE (blue lines) and CVODE (red lines) with *E. coli* database

The predicted errors of ODE and CVODE are depicted in Fig. 7, which reveal that the predicted errors of CVODE are closer to zero than ones of ODE. The predicted RMSE values of ODE and CVODE are listed in Table 4. For Gene1, Gene2, Gene4, Gene7 and Gene8, CVODE has smaller predicted RMSE values than ODE. For Gene3, Gene5 and Gene6, ODE performs better than CVODE. On average CVODE could improve 20.69% prediction accuracy and more accurately forecast gene expression data than ODE.

**Table 4:** Prediction RMSE values of ODE and CVODE for eight genes

Genes	ODE	CVODE
**Crp** (Gene 1)	0.013349	**0.010013**
**araC** (Gene 2)	0.058062	**0.031771**
**nagC** (Gene 3)	**0.01153**	0.020923
**chbC** (Gene 4)	0.041527	**0.018565**
**araE** (Gene 5)	**0.008005**	0.024344
**araA** (Gene 6)	**0.023222**	0.033139
**chbA** (Gene 7)	0.028299	**0.015565**
**chbF** (Gene 8)	0.031919	**0.016927**
Averaged	0.026989	**0.021406**

The ‘bold values’ represent the better results between two methods

**Fig. 8:**
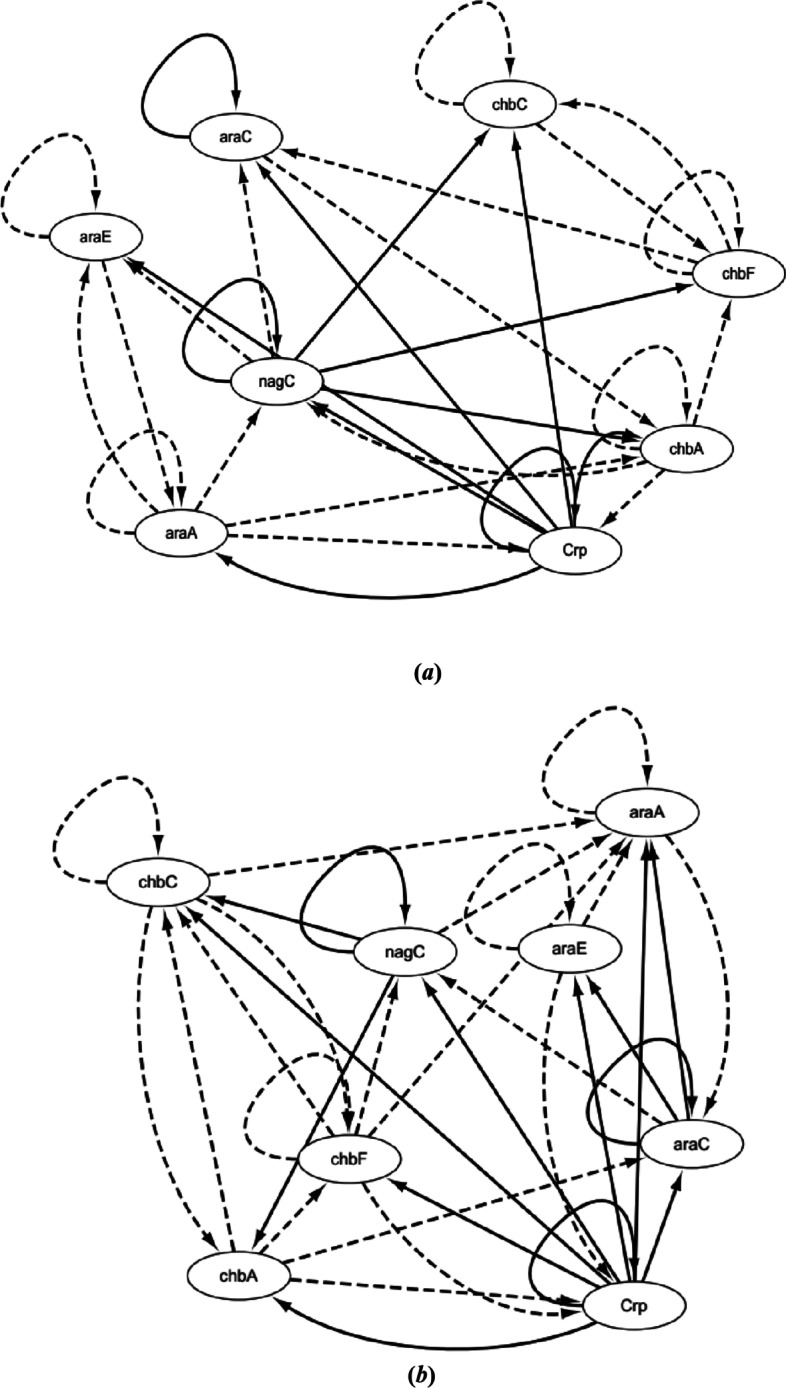
The construction *E. coli* network by ODE (**a**) and CVODE (**b**). The solid lines denote the real regulations, while the dotted lines represent the false relationships

According to the optimal CVODE models, the inferred *E. coli* sub-network is described in Fig. 8b. ODE models are also utilized to infer *E. coli* and the result is also depicted in Fig. 8a. Compared with real network structure, we can see that CVODE model could identify 14 true-positive regulations and ODE model could identify 12 true-positive relationships. The performances of $S_{n}$ and $S_{p}$ of *E.* *coli* networks inferred by ODE and CVODE are listed in Table 5. From the inferred results, it could be seen that CVODE could identify more accurately gene regulatory network than ODE model.

**Table 5:** Performances of ODE and CVODE for constructing *E. coli* network

Methods	$S_{n}$	$S_{p}$
CVODE	**0.933333**	0.591837
ODE	0.8	**0.612245**

The ‘bold values’ represent the better results between two methods

## Conclusions

In this paper, we have presented complex-valued ODE model to identify the regulations among genes for gene regulatory network inference. Grammar-guided genetic programming and complex-valued firefly algorithm are proposed to search the optimal structure and parameters of CVODE. Three real gene expression datasets are utilized and the results reveal that CVODE model could not only improve 20%-50% prediction accuracy of gene expression data, but also identify more true-positive regulatory relationships than ODE.

Our proposed method has some advantages including (1) compared with ODE, CVODE has the complex-valued structures, constants and coefficients, which could improve the modeling ability; (2) GGGP overcomes the shortcomings of GP and CFA has more population diversity and faster convergence than the traditional firefly algorithm, so our proposed hybrid optimization method based on GGGP and CFA could search the optimal model faster; (3) because of the equipment and sample tissues, the expression data may contain noise during the data collection process. Complex-valued methods have higher noise-robust memory mechanisms, so CVODE model has better performance than ODE.

In the further, CVODE will be applied for real large-scale GRN inference. The optimization of complex-valued model needs many computing resources, so the parallel technologies will improve the learning speed of model in the following work.

## Methods

### Complex-valued ordinary differential equation model

Complex-valued ordinary differential equation (CVODE) model is a variant of ODE model, whose coefficients and functions are complex-valued. Its expression is given as followed [59].

5 $$\frac{dZ}{{dt}} = \beta \cdot F(Z,t).$$

where $Z$ is complex-valued independent variable, $\beta$ is complex-valued coefficient vector, $t$ is the time point and $F( \cdot )$ is complex-valued unknown function.

### Structure optimization

Grammar-guided genetic programming (GGGP) is an improved version of genetic programming (GP), which overcomes the shortcomings of GP, such as the generation and preservation of valid programs, small search space, and complex genetic operators [51–60]. GGGP is presented to evolve the structure of CVODE model. In GGGP, context-free grammar (CFG) model is utilized to guide the evolutionary process of GP in order to obtain the optimal solution faster.

CFG model contains a quadruple, which is represented as $G = \{ N,\;T,\;P,\;\sum \}$, where $N$ is non-terminal symbol set, $T$ is terminal symbol set, $P$ is production rule set and $\sum$ is beginning symbol set. Four sets satisfy the conditions: $N \cap T = \phi$ and $\sum \in N$. An element in production rule set is represented as $x \to y$, where $x \in N$, and $y \in N \cup T$. In order to represent an example of CVODE model $\sin z + \cos z - z$, four sets of CFG model are defined in advance. $N = \{ s,\;exp,\;op,\;pre,\;var\}$, $T = \{ \sin ,\;\cos ,\; + ,\; - ,\;z\}$, $\sum = \{ s\}$, and $P$ is given as

6 $$\begin{aligned} & s \to \exp \\ & \exp \to \exp \;op\;\exp \\ & \exp \to pre\;\exp \\ & \exp \to {\text{var}} \\ & pre \to \sin |\cos \\ & op \to + | - \\ & {\text{var}} \to z. \\ \end{aligned}$$

**Fig. 9:**
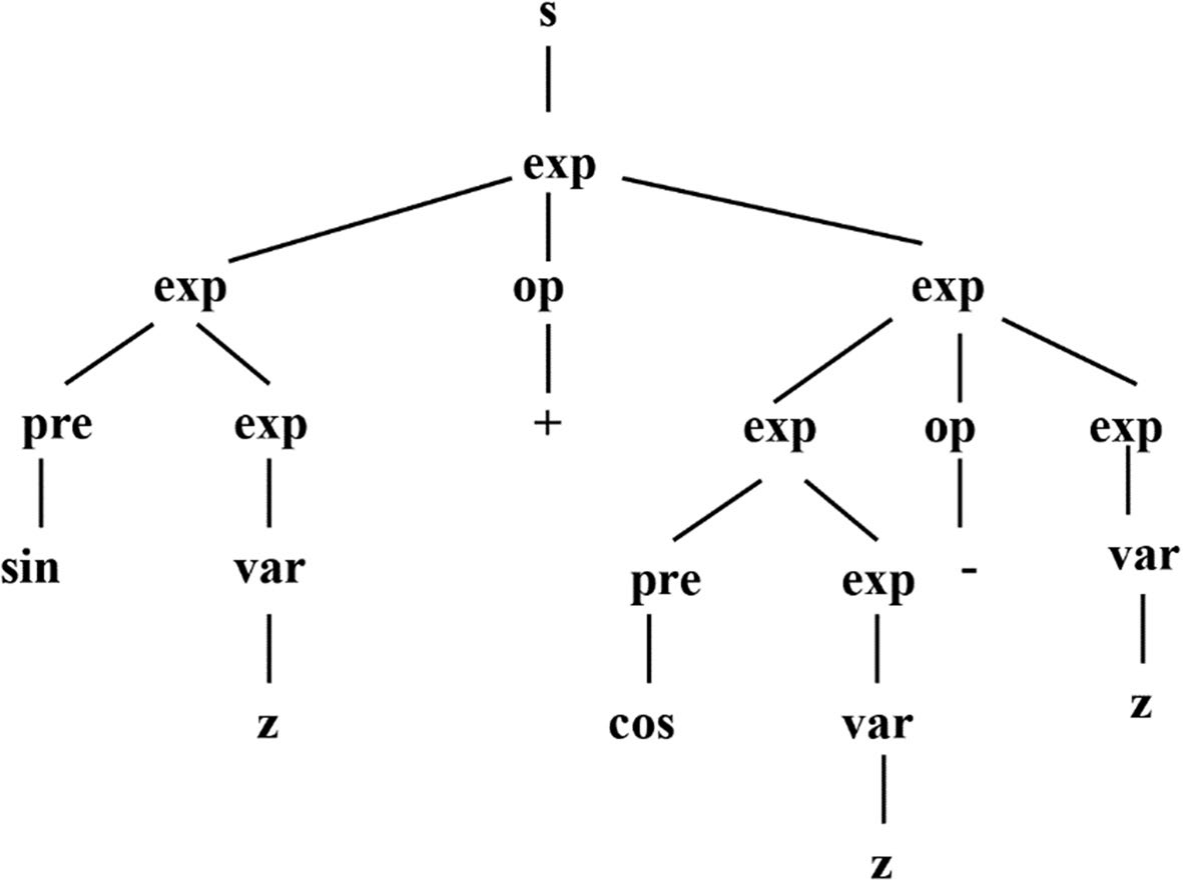
The derived tree of CVODE model $\sin z + \cos z - z$

The derived tree of CVODE model $\sin z + \cos z - z$ is obtained by generating sentence through context-free grammar, which is depicted in Fig. 9.

In GGGP, the individuals come from the derived trees of the grammar models. As the same as GP, GGGP also has genetic operators to evolve the derived trees, which contain selection, replication, crossover and mutation. Selection and replication mechanisms are consistent with GP. For crossover mechanism, two derived trees are randomly selected, and two sub-trees with the same source characteristics are selected to be crossed, whose root nodes have the same non-terminal symbols. For mutation mechanism, an internal node is selected randomly. If the node is a non-terminal node, the node is retained and the sub-tree with this node as the root node is deleted and replaced by a sub-tree created by syntax rules. If this node is the terminal node, it is replaced by a new terminal node created randomly.

### Parameter optimization

Complex-valued firefly algorithm (CFA) is an efficient complex-valued evolutionary algorithm, which is based on the mutual attraction and movement processes of complex-valued fireflies [52]. Compared with many traditional evolutionary algorithms, CFA has some advantages, such as simple design, few parameters, strong robustness, high population diversity and fast convergence.

In CFA, the positions of firefly populations are complex, so the real parts and imaginary parts of the positions of firefly populations need to be optimized in parallel. In CVODE model, complex-valued constants and coefficients need to be optimized. In this paper, CFA is presented to evolve the parameters of CVODE. The detail optimization process of parameters of CVODE with CFA is given in Algorithm 1.
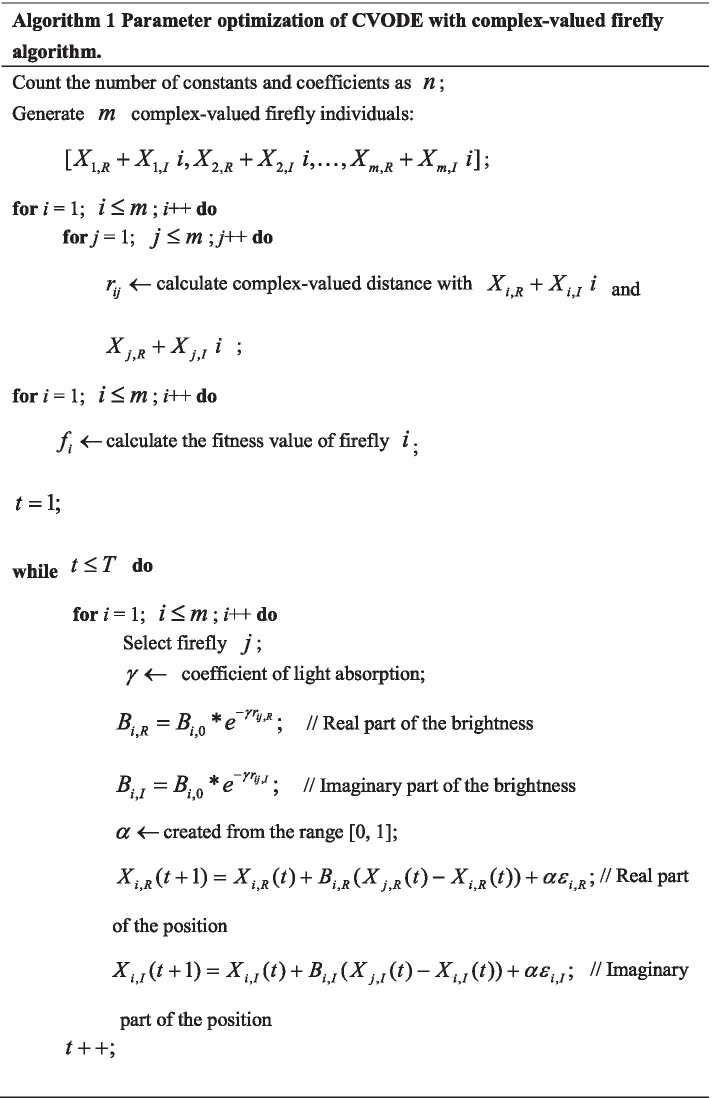


### The flowchart of GRN inference with CVODE

**Fig. 10:**
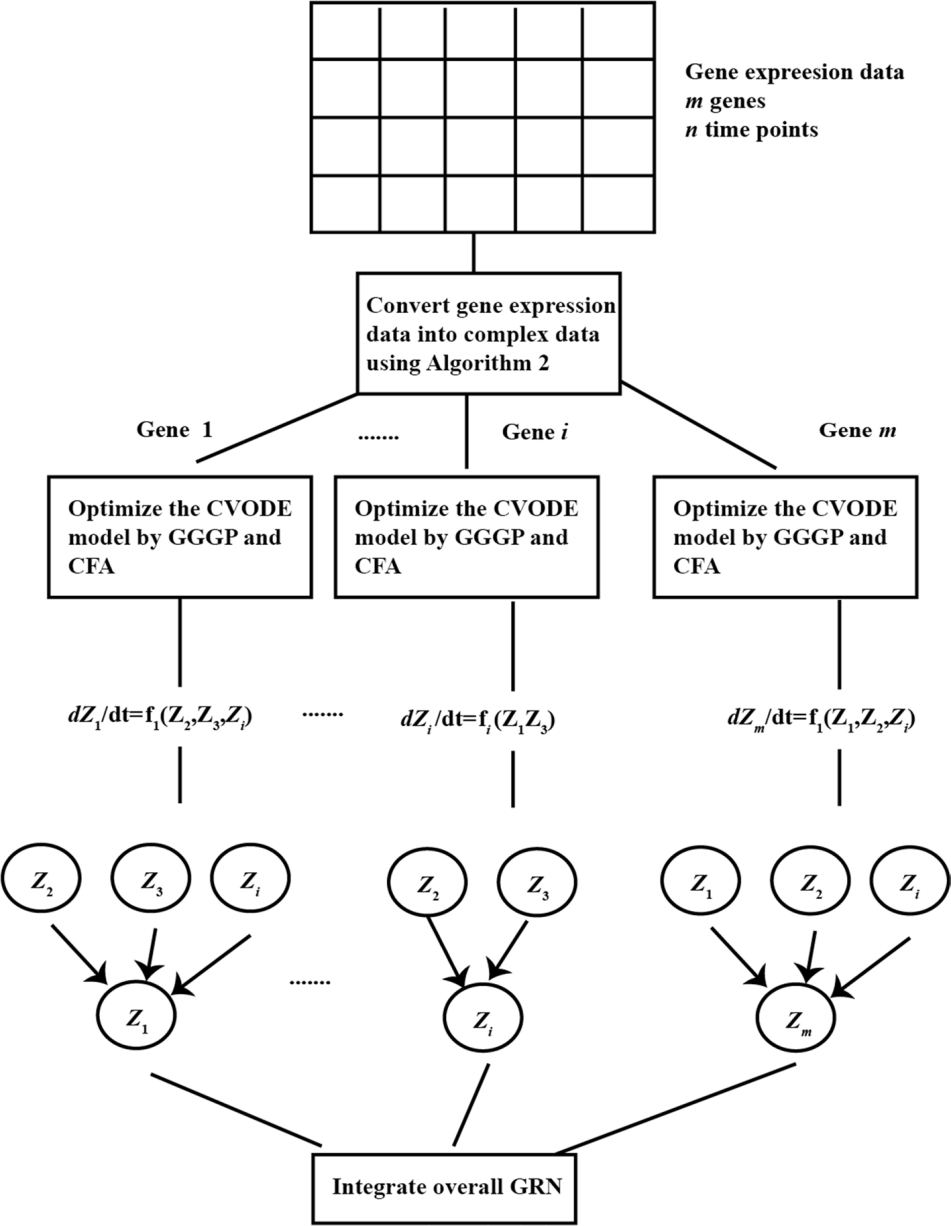
The flowchart of CVODE optimization for GRN inference


In this paper, CVODE is utilized to infer gene regulatory network, in which GGGP and CFA are proposed to optimize CVODE model. Suppose that gene expression data $[D_{1} ,D_{2} , \ldots ,D_{m} ]$ contains $m$ genes and each gene contains $n$ time points ($D_{i} = [D_{{_{i} }}^{1} ,D_{i}^{2} , \ldots ,D_{i}^{n} ]$). The inference flowchart of GRN with $m$ genes by our proposed algorithm is described in Fig. 10 The detailed process is given as follows.

1. Due to that the input data of CVODE model are complex-valued, gene expression data need to convert into complex data using Algorithm 2 before GRN inference.
2. The regulatory relationships of each target gene are inferred by CVODE model independently. For gene *i*, with gene expression data, the process of searching the optimal CVODE model is introduced as followed.
  1. Initialize $N$ CVODE individuls.
  2. Calculate the fitness values of $N$ CVODE individuals. The complex outputs need to be converted into real values by Algorithm 3.
  3. GGGP is applied to search the optimal structure of CVODE, which is introduced in Sect. 2.2. At some iterations, CFA is utilized to search the optimal complex-valued parameters of CVODE, whose structure is fixed.
  4. If the satisfied condition is achieved, the optimal process stops; otherwise, go to step 2).
3. If the optimal CVODE contains independent variables, the corresponding genes of independent variables could regulate the target gene. For example gene $j$ is included in the optimal CVODE $\frac{{dZ_{i} }}{dt} = f(Z_{j} )$, which means that gene $i$ is regulated by gene $j$.
4. $i + + .$ If the regulatory relationships of all genes have been identified, create overall gene regulatory network; otherwise go to step (2).

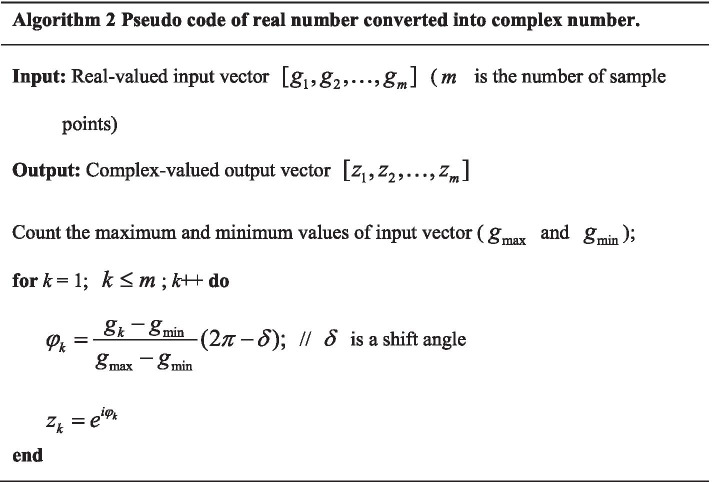



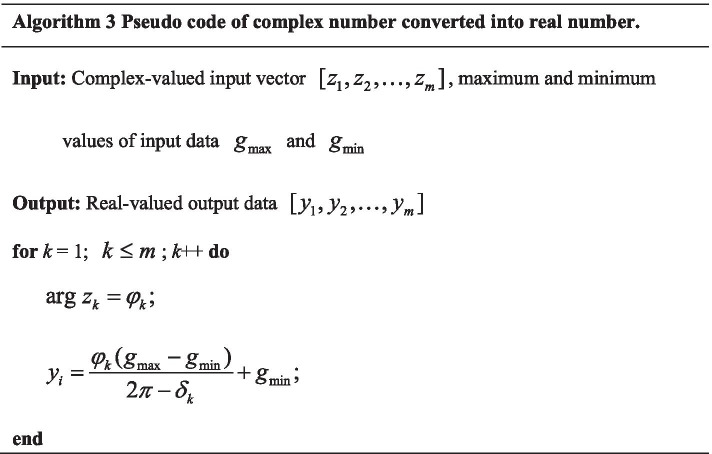


## Data Availability

Data used in this study are openly available. SOS DNA repair network data is available from http://www.weizmann.ac.il/mcb/UriAlon/download/downloadable-data. Human cell time-series data is available from http://genome-www.stanford.edu/Human-CellCycle/HeLa/. The *E. coli* data is available from http://m3d.mssm.edu/.

## Abbreviations

GRN: Gene regulatory network
CVODE: Complex-valued version of ordinary differential equation
GGGP: Grammar-guided genetic programming
ODE: Ordinary differential equation
CVRBFNN: Complex-valued radial basis function neural network
CFA: Complex-valued firefly algorithm
GP: Genetic programming
CFG: Context-free grammar
